# Cholelithiasis and Nephrolithiasis in HIV-Positive Patients in the Era of Combination Antiretroviral Therapy

**DOI:** 10.1371/journal.pone.0137660

**Published:** 2015-09-11

**Authors:** Kuan-Yin Lin, Sih-Han Liao, Wen-Chun Liu, Aristine Cheng, Shu-Wen Lin, Sui-Yuan Chang, Mao-Song Tsai, Ching-Hua Kuo, Mon-Ro Wu, Hsiu-Po Wang, Chien-Ching Hung, Shan-Chwen Chang

**Affiliations:** 1 Division of Infectious Diseases, Department of Internal Medicine, Taipei City Hospital, Kun-Ming Branch, Taipei, Taiwan; 2 Department of Internal Medicine, National Taiwan University Hospital Hsin-Chu Branch, Hsin-Chu, Taiwan; 3 Department of Internal Medicine, National Taiwan University Hospital and National Taiwan University College of Medicine, Taipei, Taiwan; 4 Department of Pharmacy, National Taiwan University Hospital, Taipei, Taiwan; 5 Graduate Institute of Clinical Pharmacy, National Taiwan University, Taipei, Taiwan; 6 Department of Laboratory Medicine, National Taiwan University Hospital, Taipei, Taiwan; 7 Department of Clinical Laboratory Sciences and Medical Biotechnology, National Taiwan University College of Medicine, Taipei, Taiwan; 8 Department of Internal Medicine, Far Eastern Memorial Hospital, New Taipei City, Taiwan; 9 School of Pharmacy, National Taiwan University, Taipei, Taiwan; 10 Department of Medical Research, China Medical University Hospital, Taichung, Taiwan; 11 China Medical University, Taichung, Taiwan; Azienda ospedaliero-universitaria di Perugia, ITALY

## Abstract

**Objectives:**

This study aimed to describe the epidemiology and risk factors of cholelithiasis and nephrolithiasis among HIV-positive patients in the era of combination antiretroviral therapy.

**Methods:**

We retrospectively reviewed the medical records of HIV-positive patients who underwent routine abdominal sonography for chronic viral hepatitis, fatty liver, or elevated aminotransferases between January 2004 and January 2015. Therapeutic drug monitoring of plasma concentrations of atazanavir was performed and genetic polymorphisms, including UDP-glucuronosyltransferase (UGT) 1A1*28 and multidrug resistance gene 1 (MDR1) G2677T/A, were determined in a subgroup of patients who received ritonavir-boosted or unboosted atazanavir-containing combination antiretroviral therapy. Information on demographics, clinical characteristics, and laboratory testing were collected and analyzed.

**Results:**

During the 11-year study period, 910 patients who underwent routine abdominal sonography were included for analysis. The patients were mostly male (96.9%) with a mean age of 42.2 years and mean body-mass index of 22.9 kg/m^2^ and 85.8% being on antiretroviral therapy. The anchor antiretroviral agents included non-nucleoside reverse-transcriptase inhibitors (49.3%), unboosted atazanavir (34.4%), ritonavir-boosted lopinavir (20.4%), and ritonavir-boosted atazanavir (5.5%). The overall prevalence of cholelithiasis and nephrolithiasis was 12.5% and 8.2%, respectively. Among 680 antiretroviral-experienced patients with both baseline and follow-up sonography, the crude incidence of cholelithiasis and nephrolithiasis was 4.3% and 3.7%, respectively. In multivariate analysis, the independent factors associated with incident cholelithiasis were exposure to ritonavir-boosted atazanavir for >2 years (adjusted odds ratio [AOR], 6.29; 95% confidence interval [CI], 1.12–35.16) and older age (AOR, 1.04; 95% CI, 1.00–1.09). The positive association between duration of exposure to ritonavir-boosted atazanavir and incident cholelithiasis was also found (AOR, per 1-year exposure, 1.49; 95% CI, 1.05–2.10). The associated factors with incident nephrolithiasis were hyperlipidemia (AOR, 3.97; 95% CI, 1.32–11.93), hepatitis B or C coinfection (AOR, 3.41; 95% CI, 1.09–10.62), and exposure to abacavir (AOR, 12.01; 95% CI, 1.54–93.54). Of 180 patients who underwent therapeutic drug monitoring of plasma atazanavir concentrations and pharmacogenetic investigations, we found that the atazanavir concentrations and UGT 1A1*28 and MDR1 G2677T/A polymorphisms were not statistically significantly associated with incident cholelithiasis and nephrolithiasis.

**Conclusions:**

In HIV-positive patients in the era of combination antiretroviral therapy, a high prevalence of cholelithiasis and nephrolithiasis was observed, and exposure to ritonavir-boosted atazanavir for >2 years was associated with incident cholelithiasis.

## Introduction

Both cholelithiasis and nephrolithiasis are widespread conditions constituting a major health burden, affecting an estimated 10–15% and 2–20% of the adult population, respectively [1]. The prevalence and incidence of cholelithiasis and nephrolithiasis vary with geographic locations and have increased over the past decades [2,3]. The increasing rates of cholelithiasis and nephrolithiasis are multifactorial, and several demographic and metabolic factors have been identified as risk factors [1]. In contrast, few studies have investigated the epidemiology of cholelithiasis and nephrolithiasis in people infected with HIV [4,5]. Previous studies have linked protease inhibitors (PIs) to cholelithiasis and nephrolithiasis, for example indinavir, a first-generation PI, which is well known for its crystallization in urine [6]. More recently, ritonavir-boosted atazanavir (atazanavir/ritonavir) has been associated with cholelithiasis and nephrolithiasis [4,7,8]. However, the impact of atazanavir/ritonavir exposure on cholelithiasis and nephrolithiasis remains difficult to estimate since screening methods using sonography were not routinely performed [9].

Modifiable risk factors of cholelithiasis and nephrolithiasis such as offending drugs are worthwhile to identify. In some circumstances, therapeutic drug monitoring (TDM) has been applied to minimize indinavir-related nephrolithiasis [10,11]. While no direct evidence of the association has been established between plasma atazanavir concentrations and cholelithiasis and nephrolithiasis, switch from atazanavir/ritonavir to unboosted atazanavir guided by TDM may reduce atazanavir-related hyperbilirubinemia [12]. On the other hand, UDP-glucuronosyltransferase (UGT) 1A1 and multidrug resistance gene 1 (MDR1) 2677 may also alter plasma atazanavir concentrations, with unknown consequences on the rate of atazanavir-induced cholelithiasis and nephrolithiasis [13,14]. In this study, we aimed to investigate the prevalence and incidence of cholelithiasis and nephrolithiasis, and to identify their associated factors among HIV-positive Taiwanese patients.

## Patients and Methods

### Ethics statement

This study was approved by the Research Ethics Committee of National Taiwan University Hospital (registration number, NTUH-201404010RIN). All patients signed written informed consent to provide their clinical and laboratory data for research before recruitment.

### Study population and study setting

This retrospective cohort study was conducted at the National Taiwan University Hospital, which is the major designated hospital for HIV care in Taiwan. HIV-positive patients were eligible for recruitment if they were aged 20 years or greater and had undergone routine abdominal sonography for chronic viral hepatitis, fatty liver, or elevated aminotransferases between January 2004 and January 2015. The sonography was performed according to routine clinical practice and not specifically for the study. Both antiretroviral-naive and-experienced patients were included, but patients with uncertain previous antiretroviral regimens and cholecystectomy prior to sonography were excluded.

According to the Taiwanese treatment guidelines for HIV infection, non-nucleoside reverse-transcriptase inhibitor (nNRTI)-containing regimens are the preferred regimens for antiretroviral-naive patients [15]. During the study period, the available PIs included nelfinavir, saquinavir, indinavir, ritonavir-boosted lopinavir, atazanavir, and darunavir.

### Data collection

The abdominal sonography was performed by certified physicians, and the hepatobiliary and genitourinary systems were completely imaged on the sonography for detection of gallstones and renal stones. All patients were followed until gallstones or renal stones were detected, they were lost to follow-up, or to 31st January, 2015 when this study terminated, whichever occurred first. Only the sonographic results after the diagnosis of HIV infection were recorded, and the first positive sonographic results or the latest negative sonographic results for gallstones or renal stones were used for analysis in patients having undergone sonography more than once.

A standardized case record form was used to collect clinical information prior to the sonography, which included demographics, body-mass index (BMI), underlying diseases, HIV-related factors such as hepatitis B or C coinfection, history and duration of antiretroviral therapy, baseline and follow-up CD4 lymphocyte counts, plasma HIV RNA loads (PVL), and laboratory data (liver-function tests, lipid profile, estimated glomerular filtration rate [GFR], serum uric acid, urine pH, and urinary crystal). We recorded the history of all prior and current antiretroviral regimens with exposure duration >3 months before abdominal sonography, and a history of exposure to nNRTIs or PIs for >2 years was regarded as a variable in logistic regression according to the previous study [4].

Chronic hepatitis B coinfection was defined as the persistence of hepatitis B virus surface antigen for >6 months, and hepatitis C coinfection was defined by positive anti-HCV antibody. Estimated GFR was calculated with the use of the Modification of Diet in Renal Diseases (MDRD) Study equation. The baseline values of CD4 lymphocyte count and PVL were obtained at the diagnosis of HIV. Sequential laboratory parameters were updated within 6 months before abdominal sonography.

### Laboratory investigations

#### CD4 lymphocyte count and plasma HIV RNA load

CD4 lymphocyte count was determined using flow cytometry (BD FACS Calibur, Becton Dickinson and Coulter Epics XL, Beckman Coulter, CA, USA). PVL was quantified using the Cobas AmpliPrep/Cobas TaqMan HIV-I test (version 2.0, Roche Molecular Systems, Inc.) with a lower detection limit of 20 copies/mL since June 2012.

#### Determination of plasma atazanavir concentrations

To optimize antiretroviral therapy, TDM of the two most commonly used antiretroviral agents, efavirenz and atazanavir, has been available at this hospital since 2009 [16]. In this study, the patients who underwent TDM of plasma atazanavir concentrations and determinations of genetic polymorphisms and had undergone sonography were included to investigate the correlation between stone formation and plasma atazanavir concentrations. We collected the closest data that antedated the date of abdominal sonography. The recommended trough plasma concentration (C _trough_) of atazanavir ranges between 0.15 and 0.85 mg/L, which is associated with virological suppression and less unconjugated hyperbilirubinemia [17]. Either mid-dosing interval (C_12_) or trough (C_24_) concentration was determined using high-performance liquid chromatography (HPLC) based on a validated method by Ramachandran et al. with minor modifications [18]. The detailed methods for determination of plasma atazanavir concentrations are available in the supplement **(S1 Text)**.

#### Genetic polymorphisms

Two genetic polymorphisms of UGT 1A1 and MDR1 2677 were determined according to previous studies with minor modifications [19,20]. Genomic DNA was extracted from peripheral blood mononuclear cell with use of the Wizard Genomic DNA purification kit (Promega, WI, USA). The TATA box of the UGT1A1 promoter and genotypes at the MDR1 locus were determined by polymerase chain reaction (PCR) amplification and further sequenced or restriction fragment-length polymorphism (RFLP) analysis, respectively. The other detailed methods for determination of genetic polymorphisms are available in the supplement **(S1 Text)**.

### Investigations of prevalence and incidence of and factors associated with cholelithiasis and nephrolithiasis

**Fig 1:**
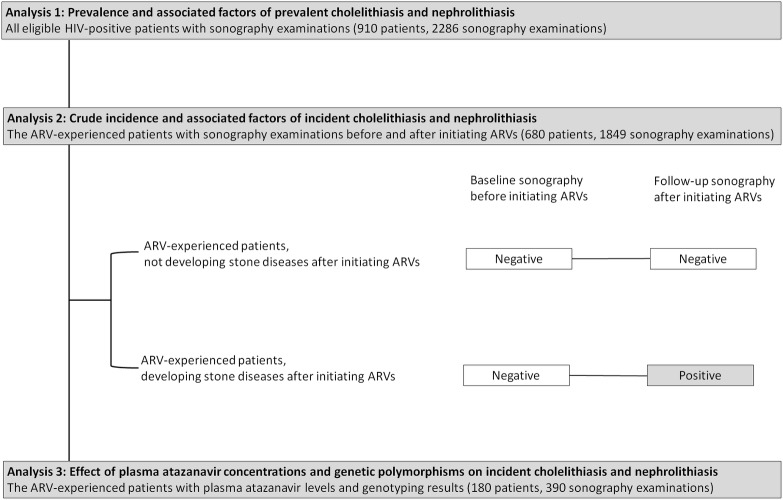
Flow diagram of patient selection for three analyses. **Abbreviation:** ARV, antiretroviral.

This study comprised separate analyses **(Fig 1)**. Analysis 1 included all eligible HIV-positive patients who had undergone routine abdominal sonography to estimate the prevalence of cholelithiasis and nephrolithiasis and their associated factors. Analysis 2 included only the antiretroviral-experienced patients who had undergone sonography before and after initiating antiretroviral therapy to determine the crude incidence of cholelithiasis and nephrolithiasis and their associated factors. Patients in analysis 2 were classified as antiretroviral-experienced patients who did not develop cholelithiasis and/or nephrolithiasis and antiretroviral-experienced patients who developed cholelithiasis and/or nephrolithiasis after therapy. Analysis 3 included only the subset of patients on atazanavir for more than 3 months with TDM and genetic polymorphism data to determine the effect of plasma atazanavir concentrations and genetic polymorphisms on the occurrence of incident cholelithiasis and nephrolithiasis.

### Statistical analysis

We used descriptive statistics to evaluate distributions of patients’ demographics and clinical characteristics. Categorical variables were compared with a chi-square test or Fisher’s exact test if the expected values were <10. Continuous variables were described as mean ± standard deviation (SD), and were compared using the Student’s *t*-test, or were described as the median and range, and were compared with the Wilcoxon rank-sum test if their distributions were not normal. All tests were two-tailed and *P* <0.05 was considered statistically significant. The crude incidence of stone disease was the rate at which new cases with cholelithiasis or nephrolithiasis occurred during the study period. Associated factors with prevalent or incident cholelithiasis and nephrolithiasis were identified using multivariate logistic regression model. Variables considered for entry into multivariate logistic regression model included variables with *P* values <0.10 in univariate analysis. 95% confidence intervals (CIs) of odds ratios (ORs) were computed to estimate the effects of each variable on cholelithiasis and nephrolithiasis. Statistical analyses were performed using SPSS software version 17.0 (SPSS Inc., Chicago, IL, USA).

## Results

### Analysis one: the prevalence and factors associated with prevalent cholelithiasis and nephrolithiasis

During the 11-year study period, we included 910 HIV-positive Taiwanese patients who had undergone abdominal sonography for chronic hepatitis B or C (464/910, 51.0%), fatty liver (343/910, 37.7%), or elevated aminotransferases (268/910, 29.5%). The total number of sonography performed among these patients was 2286, and the median interval of follow-up sonography was 329 days (interquartile range [IQR], 189–490 days). The demographics and clinical characteristics of these 910 patients are summarized in **Table 1**. Most patients were male (96.9%) with a mean age of 42.2 years and mean BMI of 22.9 kg/m^2^. Nearly half (51.0%) of the patients had had a history of chronic hepatitis B or C coinfection, for which serial follow-up abdominal sonography was performed. The major underlying diseases were hyperlipidemia (20.3%), followed by chronic hepatitis (12.6%) and hypertension (10.9%). The percentage of patients who were taking antiretroviral therapy was 85.8% (781/910), and the anchor antiretroviral agents combined with 2 NRTIs included nNRTIs (49.3%), unboosted atazanavir (34.4%), lopinavir/ritonavir (20.4%), and atazanavir/ritonavir (5.5%).

**Table 1:** The demographics and clinical characteristics of 910 HIV-positive patients who had undergone abdominal sonography.

Variable	All patients (N = 910)
**Demographics**	
Age, mean ± SD, years	42.2 ± 10.6
Male sex, n (%)	882 (96.9)
Body-mass index, mean ± SD, kg/m^2^	22.9 ± 3.5
**Underlying diseases, n (%)**	
Hyperlipidemia	185 (20.3)
Chronic hepatitis^a^	115 (12.6)
Hypertension	99 (10.9)
Diabetes mellitus	56 (6.2)
Liver cirrhosis	26 (2.9)
Chronic kidney disease	20 (2.2)
**HIV-related factors**	
Homosexual male, n (%)	711 (78.1)
Hepatitis B or C coinfection, n (%)	464 (51.0)
Duration of HIV infection, mean ± SD, years	6.4 ± 5.3
Duration of antiretroviral therapy, mean ± SD, years	4.9 ± 4.5
**History of antiretroviral therapy, n (%)**	
Antiretroviral-experienced	781 (85.8)
Zidovudine	291 (32.0)
Abacavir	422 (46.4)
Tenofovir	442 (48.6)
Other NRTIs^b^	149 (16.4)
NNRTI	449 (49.3)
Unboosted atazanavir	313 (34.4)
Atazanavir/ritonavir	50 (5.5)
Lopinavir/ritonavir	186 (20.4)
Darunavir/ritonavir	29 (3.2)
Indinavir/ritonavir	38 (4.2)
**Baseline laboratory investigations** ^**c**^	
Baseline PVL, median (range), log_10_ copies/mL	5.08 (1.94–7.23)
Baseline CD4 count, median (range), cells/μL	151.0 (0–1265.0)
**Follow-up laboratory investigations** ^**d**^	
Follow-up PVL, median (range), log_10_ copies/mL	UD (UD-7.00)
Follow-up CD4 count, median (range), cells/μL	533.0 (1.0–2091.0)

**Abbreviations:** NNRTI, non-nucleoside reverse-transcriptase inhibitor; NRTI, nucleoside reverse-transcriptase inhibitor; PVL, plasma HIV RNA load; SD, standard deviation; UD, undetectable.

^a^Chronic hepatitis was defined as persistent elevation in serum aminotransferases for 6 months or longer.

^b^Other NRTIs included stavudine, didanosine, and zalcitabine.

^c^Baseline laboratory investigations were the laboratory data obtained at the diagnosis of HIV.

^d^Follow-up laboratory investigations were the laboratory data obtained within 6 months before abdominal sonography.

**Fig 2:**
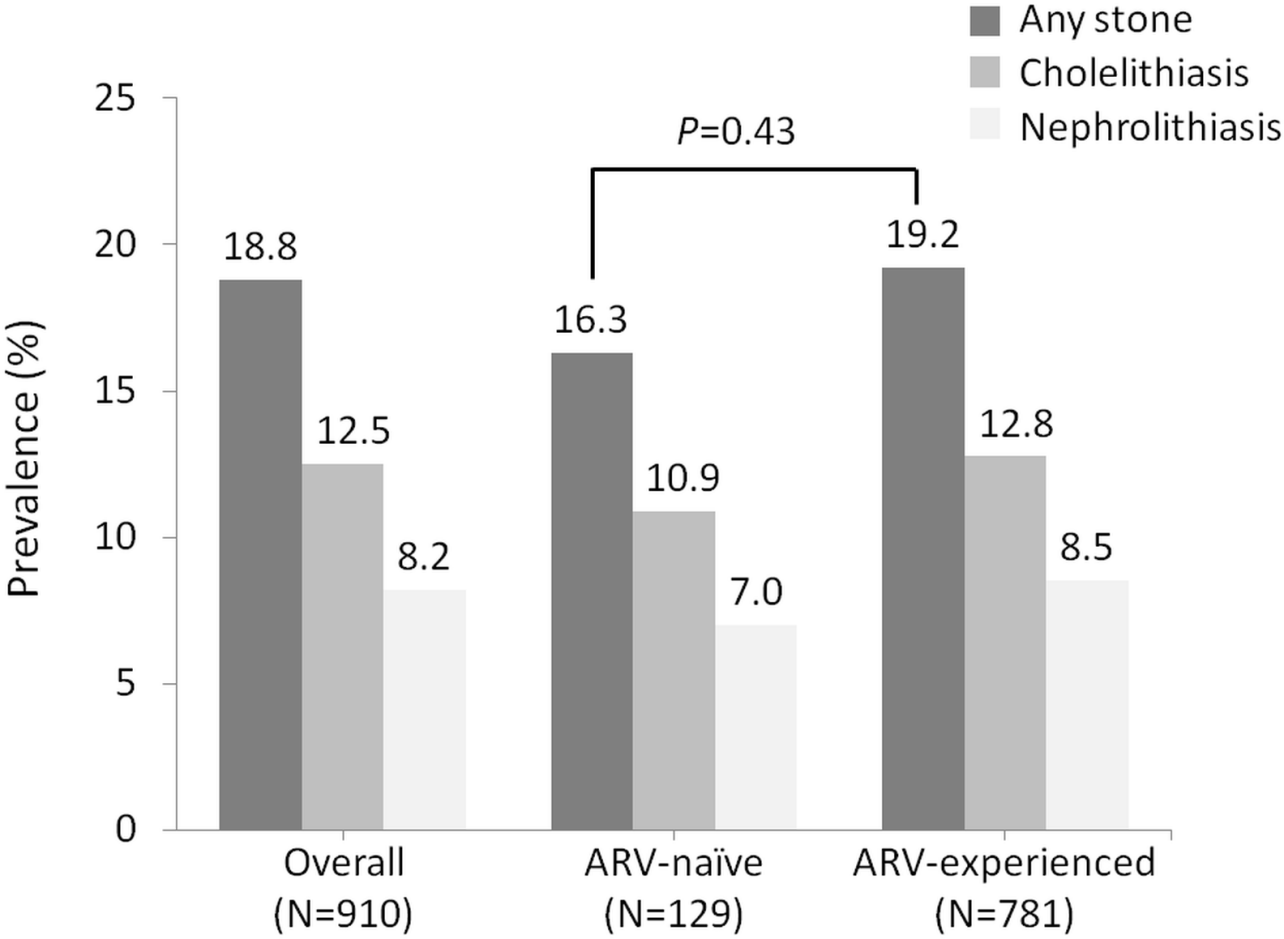
The prevalence of cholelithiasis and/or nephrolithiasis in 910 HIV-positive patients who had undergone abdominal sonography. **Abbreviation:** ARV, antiretroviral.

The overall prevalence of cholelithiasis and/or nephrolithiasis was 18.8% (171/910), which included 12.5% (114/910) for cholelithiasis and 8.2% (75/910) for nephrolithiasis. Only 19 patients (19/171, 11.1%) had subsequent complications (i.e. cholecystitis and hydronephrosis) and 16 patients received further interventions to relieve the symptoms. In antiretroviral-naïve and antiretroviral-experienced patients, the prevalence of cholelithiasis and/or nephrolithiasis was 16.3% (21/129) and 19.2% (150/781), respectively (*P* = 0.43) **(Fig 2)**. The prevalence of cholelithiasis and/or nephrolithiasis in patients with exposure to atazanavir or atazanavir/ritonavir, PIs other than atazanavir, and nNRTIs was 22.0%, 20.8%, and 18.9%, respectively (all *P*>0.05 compared with antiretroviral-naïve patients). In multivariate analysis, the independent factors associated with prevalent cholelithiasis were exposure to atazanavir/ritonavir for >2 years (adjusted OR [AOR], 4.70; 95% CI, 1.34–16.54), older age (AOR, per 1-year increase, 1.04; 95% CI, 1.01–1.06), duration of antiretroviral therapy (AOR, per 1-year increase, 0.91; 95% CI, 0.84–0.99), and elevated serum total bilirubin (AOR, per 1-mg/dL increase, 1.29; 95% CI, 1.05–1.57) **(S1 Table)**. For nephrolithiasis, the associated factors were increasing age (AOR, per 1-year increase, 1.03; 95% CI, 1.00–1.06), decreased estimated GFR (AOR, per 1-ml/min/1.73m^2^ decrease, 1.02; 95% CI, 1.01–1.03), and elevated serum total cholesterol (AOR, per 1-mg/dL increase, 1.01; 95% CI, 1.01–1.02) **(S1 Table)**.

### Analysis two: crude incidence and factors associated with incident cholelithiasis and nephrolithiasis

A total of 680 antiretroviral-experienced patients who had undergone sonography before and after initiating antiretroviral therapy were included to estimate the crude incidences of cholelithiasis and nephrolithiasis. The total number of sonography performed among these patients was 1849, and the median interval of follow-up sonography was 332 days (IQR, 189–492 days). The indications of sonography were hepatitis B or C (346/680, 50.9%), fatty liver (240/680, 35.3%), or elevated aminotransferases (142/680, 20.9%). The demographics and clinical characteristics of patients with or without incident cholelithiasis and nephrolithiasis are shown in **Table 2**. The cumulative crude incidence of developing cholelithiasis and/or nephrolithiasis was 7.2% (4 9/680), which included 4.3% (29/680) for cholelithiasis and 3.7% (25/680) for nephrolithiasis after initiating antiretroviral therapy. The crude incidence in patients receiving regimens containing nNRTI, unboosted/boosted atazanavir, and PIs other than atazanavir was 7.6%, 8.8%, and 6.8%, respectively. The crude incidences were not statistically different among the patients receiving different antiretroviral regimens (*P*>0.05).

**Table 2:** The demographics and clinical characteristics of 680 antiretroviral-experienced patients with or without incident cholelithiasis and nephrolithiasis.

	Cohort 1^a^			Cohort 2^a^		
Variable	Cholelithiasis (N = 29)	No cholelithiasis (N = 651)	*P*	Nephrolithiasis (N = 25)	No nephrolithiasis (N = 655)	*P*
**Demographics**						
Age, mean ± SD, years	47.4 ± 9.8	42.1 ± 10.7	0.002	48.6 ± 12.4	42.1 ± 10.5	0.001
Male sex, n (%)	27 (93.1)	635 (97.5)	0.18	23 (92.0)	639 (97.6)	0.14
Body-mass index, mean ± SD, kg/m^2^	23.9 ± 3.4	22.8 ± 3.4	0.17	22.1 ± 3.4	22.9 ± 3.3	0.13
**Underlying diseases, n (%)**						
Hyperlipidemia	7 (24.1)	133 (20.4)	0.64	9 (36.0)	131 (20.0)	0.07
Chronic hepatitis^b^	7 (24.1)	71 (10.9)	0.04	5 (20.0)	73 (11.1)	0.19
Hypertension	4 (13.8)	60 (9.2)	0.34	6 (24.0)	58 (8.9)	0.02
Diabetes mellitus	3 (10.3)	31 (4.8)	0.17	1 (4.0)	33 (5.0)	0.99
Liver cirrhosis	1 (3.4)	17 (2.6)	0.55	1 (4.0)	17 (2.6)	0.50
Chronic kidney disease	1 (3.4)	13 (2.0)	0.46	2 (8.0)	12 (1.8)	0.09
**HIV-related factors**						
Homosexual male, n (%)	23 (79.3)	523 (80.3)	0.82	17 (68.0)	529 (80.8)	0.13
Hepatitis B or C coinfection, n (%)	19 (65.5)	327 (50.2)	0.11	18 (72.0)	328 (50.1)	0.04
Duration of HIV infection, mean ± SD, years	8.3 ± 4.4	7.0 ± 5.2	0.07	9.5 ± 5.7	7.0 ± 5.1	0.02
Duration of antiretroviral therapy, mean ± SD, years	6.8 ± 3.8	5.6 ± 4.2	0.05	7.6 ± 5.0	5.6 ± 4.1	0.04
**History of antiretroviral therapy, n (%)**						
Zidovudine	11 (37.9)	234 (35.9)	0.83	11 (44.0)	234 (35.7)	0.40
Abacavir	20 (69.0)	345 (53.0)	0.13	19 (76.0)	346 (52.8)	0.03
Tenofovir	14 (48.3)	377 (57.9)	0.30	11 (44.0)	380 (58.0)	0.16
NNRTI	17 (58.6)	377 (57.9)	0.94	16 (64.0)	378 (57.7)	0.68
Duration of exposure, mean ± SD, years	2.5 ± 3.2	2.5 ± 3.4	0.88	4.3 ± 4.5	2.4 ± 3.4	0.06
> 2 years, n (%)	11 (37.9)	242 (37.2)	0.93	14 (56.0)	239 (36.5)	0.05
Unboosted atazanavir	16 (55.2)	250 (38.4)	0.07	11 (44.0)	255 (38.9)	0.61
Duration of exposure, mean ± SD, years	1.9 ± 2.3	1.3 ± 2.3	0.06	1.2 ± 1.8	1.3 ± 2.3	0.79
> 2 years, n (%)	12 (41.4)	167 (25.7)	0.06	6 (24.0)	173 (26.4)	0.99
Atazanavir/ritonavir	4 (13.8)	41 (6.3)	0.12	1 (4.0)	44 (6.7)	0.99
Duration of exposure, mean ± SD, years	0.5 ± 1.4	0.1 ± 0.6	0.09	0.04 ± 0.2	0.1 ± 0.7	0.58
> 2 years, n (%)	3 (10.3)	10 (1.5)	0.02	0 (0)	13 (2.0)	0.99
Lopinavir/ritonavir	7 (24.1)	153 (23.5)	0.99	5 (20.0)	155 (23.7)	0.81
Duration of exposure, mean ± SD, years	1.3 ± 2.8	0.9 ± 2.1	0.75	1.2 ± 2.9	0.9 ± 2.1	0.85
> 2 years, n (%)	6 (20.7)	110 (16.9)	0.61	5 (20.0)	111 (16.9)	0.60
Darunavir/ritonavir	1 (3.4)	25 (3.8)	0.99	0 (0)	26 (4.0)	0.62
Duration of exposure, mean ± SD, years	± 0.3	0.1 ± 0.3	0.92	0	0.1 ± 0.3	0.31
> 2 years, n (%)	0 (0)	8 (1.2)	0.99	0 (0)	8 (1.2)	0.99
Indinavir/ritonavir	2 (6.9)	26 (4.0)	0.34	1 (4.0)	27 (4.1)	0.99
Duration of exposure, mean ± SD, years	0.3 ± 1.3	0.2 ± 1.2	0.45	0.2 ± 1.1	0.2 ± 1.2	0.97
> 2 years, n (%)	1 (3.4)	22 (3.4)	0.99	1 (4.0)	22 (3.4)	0.58
**Baseline laboratory investigations** ^**c**^						
Baseline PVL, median (range), log_10_ copies/mL	5.42 (4.14–7.00)	5.05 (1.94–7.00)	0.05	5.35 (3.91–5.96)	5.08 (1.94–7.00)	0.21
Baseline CD4 count, median (range), cells/μL	67.0 (1–591.0)	157.0 (0–1265.0)	0.22	84.0 (0–448.0)	155.0 (0–1265.0)	0.10
**Follow-up laboratory investigations** ^**d**^						
Follow-up PVL, median (range), log_10_ copies/mL	UD (UD-4.91)	UD (UD-5.27)	0.07	UD (UD-2.96)	UD (UD-5.27)	0.41
Follow-up CD4 count, median (range), cells/μL	502.0 (4.0–1316.0)	565.0 (8.0–1965.0)	0.32	458.5 (76.0–1012.0)	565.0 (4.0–1965.0)	0.27
Estimated GFR, mean ± SD, mL/min/1.73m^2^	92.5 ± 19.5	99.1 ± 21.6	0.12	93.0 ± 32.5	99.1 ± 21.0	0.33
Serum total bilirubin, mean ± SD, mg/dL	2.2 ± 3.9	1.2 ± 1.0	0.25	1.4 ± 1.7	1.2 ± 1.2	0.57
Serum ALT, mean ± SD, U/L	49.1 ± 86.3	34.5 ± 37.9	0.25	31.3 ± 24.7	35.3 ± 41.6	0.93
Serum total cholesterol, mean ± SD, mg/dL	173.7 ± 35.4	171.6 ± 35.1	0.72	189.3 ± 40.5	171.1 ± 34.7	0.02
Serum triglyceride, mean ± SD, mg/dL	163.0 ± 104.6	155.5 ± 129.6	0.37	170.0 ± 82.8	155.3 ± 130.0	0.10
Serum uric acid, mean ± SD, mg/dL	NA	NA	NA	4.9 ± 0.0	6.2 ± 2.0	0.39
Urine pH, mean ± SD, unit	NA	NA	NA	6.2 ± 0.6	6.0 ± 0.6	0.10
Urinary crystal, n (%)	NA	NA	NA	0 (0)	15 (2.3)	0.99

**Abbreviations:** ALT, alanine aminotransferase; GFR, glomerular filtration rate; NNRTI, non-nucleoside reverse-transcriptase inhibitor; NRTI, nucleoside reverse-transcriptase inhibitor; PVL, plasma HIV RNA load; SD, standard deviation; UD, undetectable.

^a^Cohort 1 was the cohort for the comparison between patients with and those without incident cholelithiasis, and cohort 2 was the cohort for the comparison between patients with and those without incident nephrolithiasis.

^b^Chronic hepatitis was defined as persistent elevation in serum aminotransferases for 6 months or longer.

^c^Baseline laboratory investigations were the laboratory data obtained at the diagnosis of HIV.

^d^Follow-up laboratory investigations were the laboratory data obtained within 6 months before abdominal sonography.

In univariate analysis, the factors associated with incident cholelithiasis were advanced age, chronic hepatitis, exposure to atazanavir/ritonavir for >2 years, higher baseline or follow-up PVL, and elevated serum total bilirubin **(S2 Table)**. For incident nephrolithiasis, the associated factors were advanced age, hypertension, hepatitis B or C coinfection, longer duration of HIV infection or antiretroviral therapy, exposure to abacavir, and elevated serum total cholesterol (all *P*<0.05) **(S2 Table)**. In multivariate analysis, the independent factors associated with incident cholelithiasis included exposure to atazanavir/ritonavir for >2 years (AOR, 6.29; 95% CI, 1.12–35.16; *P* = 0.04) and advanced age (AOR, per 1-year increase, 1.04; 95% CI, 1.00–1.09; *P* = 0.047) **(Table 3)**. If we considered atazanavir/ritonavir exposure as a continuous variable, a positive association between the cumulative exposure and incident cholelithiasis was also found (AOR, per 1-year exposure, 1.49; 95% CI, 1.05–2.10; *P* = 0.02). For nephrolithiasis, the associated factors were hyperlipidemia (AOR, 3.97; 95% CI, 1.32–11.93; *P* = 0.01), hepatitis B or C coinfection (AOR, 3.41; 95% CI, 1.09–10.62; *P* = 0.04), and exposure to abacavir (AOR, 12.01; 95% CI, 1.54–93.54; *P* = 0.02) **(Table 4)**. There were no cases of nephrolithiasis among those exposed to atazanavir/ritonavir for >2 years or those exposed to darunavir/ritonavir. The cumulative exposure of atazanavir/ritonavir was also not statistically significantly correlated with incident nephrolithiasis.

**Table 3:** Multivariate logistic analysis to identify the factors associated with incident cholelithiasis in 680 antiretroviral-experienced patients.

Variable^a^	Cholelithiasis		
	OR	95% CI	*P*
Age, per 1-year increase	1.04	1.00–1.09	0.047
Chronic hepatitis	0.74	0.07–7.67	0.80
Unboosted atazanavir, > 2 years	1.42	0.44–4.53	0.56
Atazanavir/ritonavir, > 2 years	6.29	1.12–35.16	0.04
Baseline PVL, per 1-log_10_ copies/mL increase	1.97	0.92–4.21	0.08
Follow-up PVL, per 1-log_10_ copies/mL increase	1.22	0.76–1.96	0.40
Serum total bilirubin, per 1-mg/dL increase	1.19	0.78–1.82	0.42
Serum ALT, per 1-U/L increase	1.00	0.96–1.02	0.43

**Abbreviations:** ALT, alanine aminotransferase; CI, confidence interval; OR, odds ratio; PVL, plasma HIV RNA load.

^a^Variables considered for entry into multivariate logistic regression model included variables with *P* values <0.10 in univariate analysis.

**Table 4:** Multivariate logistic analysis to identify the factors associated with incident nephrolithiasis in 680 antiretroviral-experienced patients.

Variable^a^	Nephrolithiasis		
	OR	95% CI	*P*
Age, per 1-year increase	1.03	0.98–1.08	0.21
Hyperlipidemia	3.97	1.32–11.93	0.01
Hypertension	0.48	0.07–3.43	0.47
Chronic kidney disease	2.99	0.28–32.25	0.37
Hepatitis B or C coinfection	3.41	1.09–10.62	0.04
Duration of HIV infection, per 1-year increase	1.08	0.96–1.23	0.21
Duration of antiretroviral therapy, per 1-year increase	0.91	0.78–1.07	0.25
Use of abacavir	12.01	1.54–93.54	0.02
NNRTI exposure > 2 years	1.14	0.34–3.78	0.83
Serum total cholesterol, per 1-mg/dL increase	1.00	0.99–1.02	0.62
Urine pH, per 1-unit increase	2.31	0.98–5.45	0.06

**Abbreviations:** CI, confidence interval; NNRTI, non-nucleoside reverse-transcriptase inhibitor; OR, odds ratio.

^a^Variables considered for entry into multivariate logistic regression model included variables with *P* values <0.10 in univariate analysis.

### Analysis three: effects of plasma atazanavir concentrations and genetic polymorphisms on incident cholelithiasis and nephrolithiasis

**Fig 3:**
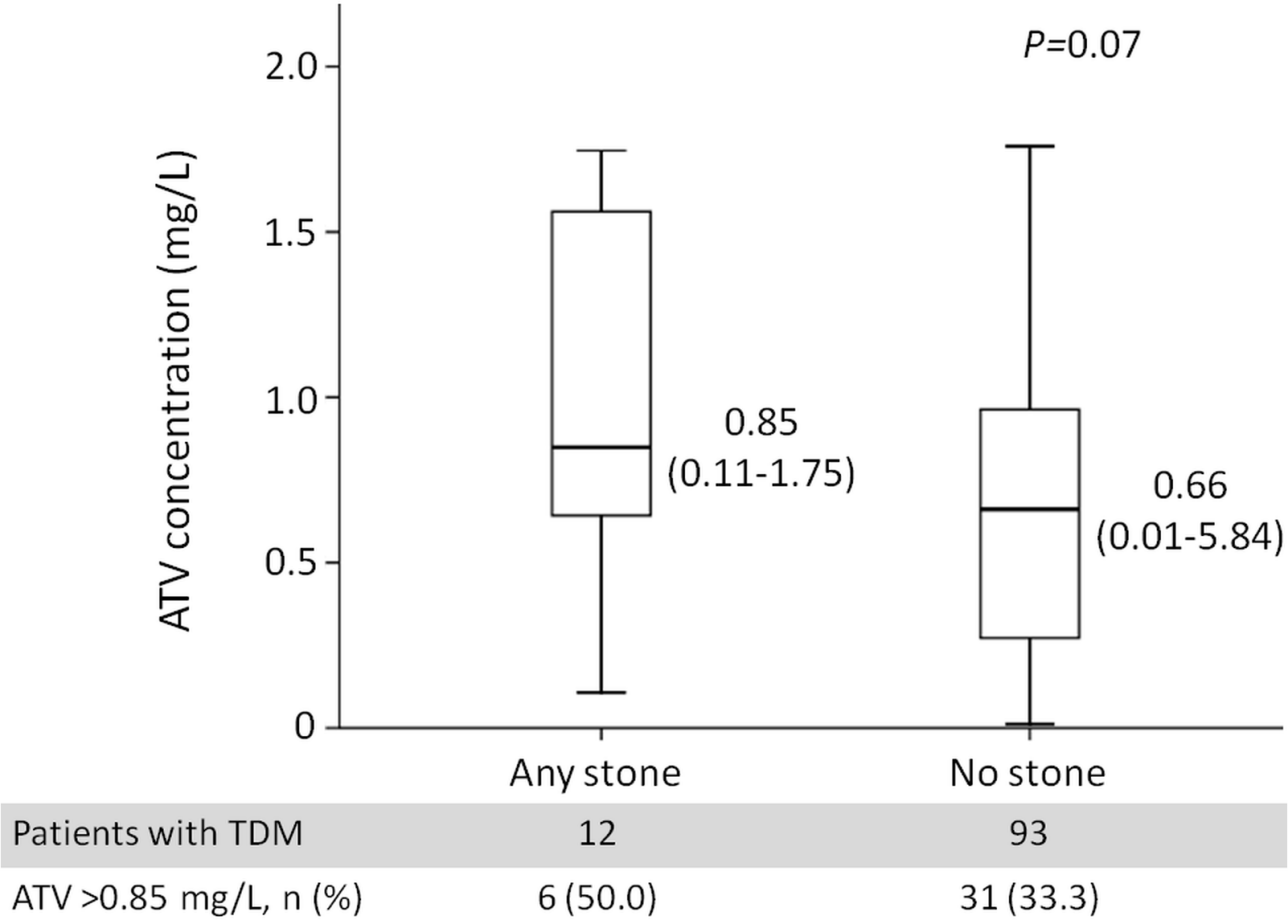
Plasma atazanavir concentrations (C_12_) of patients with (N = 12) and without (N = 93) incident cholelithiasis and/or nephrolithiasis. **Abbreviations:** ATV, atazanavir; TDM, therapeutic drug monitoring.

A total of 180 patients underwent TDM of plasma atazanavir concentrations, including 16 patients who developed cholelithiasis and/or nephrolithiasis and 164 patients who did not. The total number of sonography performed among these patients was 390, and the median interval of follow-up sonography was 375 days (IQR, 189–734 days). The indications of sonography were hepatitis B or C (65/180, 36.1%), fatty liver (81/180, 45.0%), or elevated aminotransferases (36/180, 20.0%). The atazanavir C_12_ and C_24_ were compared separately. **Fig 3** shows the comparison of atazanavir C_12_ between 12 patients with and 93 patients without incident cholelithiasis and/or nephrolithiasis. The atazanavir C_12_ was higher (median, 0.85 mg/L; range, 0.11–1.75) in patients with incident stones compared with patients without stones (median, 0.66 mg/L; range, 0.01–5.84), but the difference was of borderline significance (*P* = 0.07). The percentage of patients with atazanavir C_12_ above 0.85 mg/L, the upper limit of the therapeutic window, was 50.0% (6/12) and 33.3% (31/93) in patients with and without incident stones, respectively (*P* = 0.34). Only 4 patients with incident stones had atazanavir C_24_ levels, which were lower than that in patients without stones, but the difference was not statistically significant (*P* = 0.25).

The genotyping results of UGT1A1*28 and MDR1 2677, which may affect atazanavir metabolism and lead to hyperbilirubinemia, are shown in **Table 5**. The frequency of the UGT1A1*28 allele in 16 patients with and 161 patients without incident stones was 12.5% and 19.9%, respectively (*P* = 0.74) and that of the MDR1 G2677T/A in 7 patients with and 86 patients without incident stones was 71.4% and 70.9%, respectively (*P* = 0.99).

**Table 5:** Comparisons of genotype frequencies for UGT1A1 and MDR1 2677 between patients with and without incident cholelithiasis and/or nephrolithiasis.

Genotype	Any stonen/N (%)^a^	No stone n/N (%)^a^	*P*
UGT1A1*28			
TA6/TA6	14/16 (87.5)	129/161 (80.1)	0.74
TA6/TA7	2/16 (12.5)	32/161 (19.9)	
**MDR1 2677**			
G/G	2/7 (28.6)	25/86 (29.1)	0.99
G/T	2/7 (28.6)	29/86 (33.7)	
T/T	1/7 (14.3)	12/86 (14.0)	
T/A	1/7 (14.3)	6/86 (7.0)	
G/A	1/7 (14.3)	13/86 (15.1)	
A/A	0/7 (0)	1/86 (1.2)	
MDR1 2677 (T or A)	5/7 (71.4)	61/86 (70.9)	0.99

**Abbreviations:** MDR1, multidrug resistance gene 1; UGT, UDP-glucuronosyltransferase.

^a^N was the total number of patients with determinations of UGT1A1*28 or MDR1 2677 polymorphisms, and n was the number of patients with specific genotypes.

## Discussion

This study demonstrates a high prevalence of cholelithiasis and nephrolithiasis in the HIV-positive Taiwanese population receiving routine abdominal sonography for chronic viral hepatitis and other indications. Exposure to atazanavir/ritonavir for over 2 years is associated with a 6.29-fold increase in the risk for incident cholelithiasis. However, plasma atazanavir concentrations and genetic polymorphisms related to metabolism of atazanavir are not identified as associated factors, perhaps due to insufficient sample size. To the best of our knowledge, this study is the first analysis that attempts to elucidate the correlation between plasma atazanavir concentrations and stone formation using routine follow-up sonography.

The prevalence and incidence of cholelithiasis and nephrolithiasis are enormously affected by geography and ethnicity, and are relatively lower in Asian countries compared with Western countries [2,3]. The prevalence and incidence of gallstones have been reported to be 4.6–10.7% and 6.32 per 1000 person-years, respectively, in the general Asian population [21,22], with the prevalence and incidence of renal stones being 7.4% in the Taiwanese population, and 1.14 per 1000 person-years in the Japanese population [23,24]. In the HIV-positive population, recent studies conducted in Japan reported higher rates of cholelithiasis (9.8%) and nephrolithiasis (7.1%) by means of clinical diagnosis and imaging performed for various indications [4,7]. To avoid overestimation, we enrolled patients who underwent routine abdominal sonography since sonography has been accepted as the initial imaging modality of choice for the detection of gallstones and renal stones [2,25]. Our findings, similar to those previously reported by Japanese investigators [4,7], also showed a high prevalence of cholelithiasis (12.5%) and nephrolithiasis (8.2%), which was previously attributed to antiretrovirals, especially exposure to PIs [4,6,7,8]. In our study, the high rates of cholelithiasis and nephrolithiasis were found both in antiretroviral-naïve and antiretroviral-experienced patients without statistically significant difference, suggesting that there may be other contributing factors in addition to antiretrovirals in the HIV-positive population. Because the majority of our patients had chronic hepatitis B or C infection (51.0%), the high rates in antiretroviral-naïve patients may result from the impact of hepatitis C coinfection, which has been recognized as a risk factor for cholelithiasis [26]. Whether HIV infection itself may contribute to cholelithiasis and nephrolithiasis warrants further investigations.

Some important risk factors for cholelithiasis and nephrolithiasis have been identified previously [2,27], such as older age, female sex, metabolic syndrome, liver diseases, specific diet and drugs for cholelithiasis and male sex, metabolic syndrome, reduced urinary volume and urine pH, hyperuricosuria, and specific diet and drugs for nephrolithiasis. Among the HIV-infected patients, previously identified risk factors were hepatitis B or C coinfection and exposure to PIs, including indinavir and atazanavir [6,28,29]. Atazanavir-induced cholelithiasis and nephrolithiasis were observed in patients with cumulative exposure to atazanavir/ritonavir for >2 years [4,30–32]. These findings were based on the clinical practice that most patients received atazanavir/ritonavir as their PI-based regimens. In our study, a substantial proportion of the patients received unboosted atazanavir (34.4%) instead of atazanavir/ritonavir (5.5%). The independent factors associated with incident cholelithiasis were exposure to atazanavir/ritonavir for >2 years and older age. Our findings indicate that patients with cumulative exposure to atazanavir/ritonavir, rather than unboosted atazanavir, had a 6.29-fold increase in the risk for incident cholelithiasis. In contrast, our study failed to correlate nephrolithiasis with cumulative exposure to atazanavir/ritonavir. A recent retrospective study using insurance databases also showed no evidence of an increased risk of nephrolithiasis among patients on atazanavir compared with other PIs, but a positive association was observed when atazanavir was compared with PI-free regimens [33]. While no previous studies have ever identified an association between exposure to abacavir and incident nephrolithiasis, the positive association found in our study suggests the direct effect of abacavir or the fact that patients with decreased estimated GFR were more likely to receive abacavir. More studies are needed to confirm our findings.

Two mechanisms are hypothesized for atazanavir-induced cholelithiasis and nephrolithiasis, including precipitation of atazanavir in the less acidic bile and urine and atazanavir-related hyperbilirubinemia facilitating the formation of gallstones [29,34]. To elucidate the effect of atazanavir exposure, plasma atazanavir concentrations and genetic polymorphisms altering the metabolism and transportation of atazanavir need to be examined. To date, only one recent study addressed the relationship between genetic polymorphisms of UGT1A-3’-UTR and atazanavir-induced nephrolithiasis [13]. We examined two well-known genetic polymorphisms of UGT1A1*28 and MDR1 2677 related to atazanavir-induced hyperbilirubinemia [14], but failed to demonstrate statistically significant correlation between the genetic polymorphisms and incident cholelithiasis and nephrolithiasis. In terms of the plasma atazanavir concentrations, atazanavir C_12_ was numerically higher in patients with incident cholelithiasis and nephrolithiasis without reaching statistical significance, which is likely caused by the small sample size in our study. The minimum sample size in each group to detect a difference in atazanavir levels with a power of 80% at 95% confidence level estimated by STPLAN 4.5 would be 100. While our results should be interpreted with caution, our findings suggest that cumulative atazanavir exposure has more impact than plasma drug levels on stone formation.

Our study has several limitations. First, the indications for sonography in our study may cause overestimation of the prevalence and incidence of stone formation. On the other hand, the sensitivity of abdominal sonography for cholelithiasis and nephrolithiasis has been reported to be 97% and 54%, respectively, meaning that some stones especially ureteral stones may be missed by sonography [25, 35]. Our results may not be generalizable to those patients who did not meet the indications for sonography. Second, the follow-up of abdominal sonography was not performed at regular intervals, which made it difficult for us to estimate the incidence rates in person-years. In addition, patients without baseline abdominal sonography prior to initiation of antiretroviral therapy might cause overestimates of prevalence in treatment-experienced patients. In this study, we used crude incidence instead and excluded patients without baseline sonography from the analysis for identifying associated factors. Third, 180 patients with atazanavir exposure had determinations of plasma atazanavir concentrations and genetic polymorphisms and only 16 patients had cholelithiasis and/or nephrolithiasis. The small sample size may preclude us from identifying correlations between plasma atazanavir concentrations, genetic polymorphisms, and cholelithiasis and nephrolithiasis. Fourth, the information on the previous history of cholelithiasis and nephrolithiasis, diet and other medications except antiretroviral therapy that could contribute to cholelithiasis and nephrolithiasis may not have been recorded in the medical records, and the stone composition was not known. Therefore, we were not able to rule out other contributing factors. Lastly, our study included only Taiwanese and the results may not be generalizable to HIV-positive patients of other ethnicities in other geographic locations.

In conclusion, in HIV-positive Taiwanese patients who had undergone routine abdominal sonography for chronic viral hepatitis and other indications, a high prevalence of cholelithiasis and nephrolithiasis was observed. The cumulative exposure to atazanavir/ritonavir for >2 years was associated with incident cholelithiasis. With the limitation of insufficient sample size, we failed to demonstrate statistically significant associations between plasma atazanavir concentrations, genetic polymorphisms altering atazanavir metabolism and incident cholelithiasis and nephrolithiasis.

## Supporting Information

S1 Table Logistic analysis to estimate the factors associated with prevalent cholelithiasis and nephrolithiasis in 910 HIV-positive patients who had undergone abdominal sonography. (DOCX)

S2 Table Univariate logistic analysis to estimate the factors associated with incident cholelithiasis and nephrolithiasis in 680 antiretroviral-experienced patients. (DOCX)

S1 Text The detailed methods for determination of plasma atazanavir concentrations and genetic polymorphisms. (DOCX)
