# Supplementary material for: Cholelithiasis and Nephrolithiasis in HIV-Positive Patients in the Era of Combination Antiretroviral Therapy
Source: PLoS One. 2015 Sep 11;10(9):e0137660. doi: 10.1371/journal.pone.0137660 (PMC4567270; doi:10.1371/journal.pone.0137660)
Supplement: S1 Table — (DOCX) [file pone.0137660.s001.docx]

**Supporting information**

**S1 Table.** Logistic analysis to estimate the factors associated with prevalent cholelithiasis and nephrolithiasis in 910 HIV-positive patients who had undergone abdominal sonography.

(A) Cholelithiasis

| **Variable** | **Univariate analysis** | | | **Multivariate analysis^a^** | | |
| --- | --- | --- | --- | --- | --- | --- |
|  | **OR** | **95% CI** | ***P*** | **OR** | **95% CI** | ***P*** |
| **Demographics** |  |  |  |  |  |  |
| Age, per 1-year increase | 1.04 | 1.02-1.06 | 0.001 | 1.04 | 1.01-1.06 | 0.01 |
| Male sex | 0.29 | 0.13-0.65 | 0.003 | 0.39 | 0.09-1.79 | 0.23 |
| Body-mass index, per 1-kg/m^2^ increase | 1.05 | 0.99-1.11 | 0.14 | - | - | - |
| **Underlying diseases** |  |  |  |  |  |  |
| Hyperlipidemia | 1.82 | 1.17-2.82 | 0.01 | 1.15 | 0.54-2.42 | 0.72 |
| Chronic hepatitis | 1.69 | 1.00-2.84 | 0.05 | 1.02 | 0.38-2.71 | 0.97 |
| Hypertension | 3.54 | 2.17-5.76 | 0.001 | 1.61 | 0.69-3.83 | 0.27 |
| Diabetes mellitus | 2.79 | 1.49-5.23 | 0.001 | 0.57 | 0.13-2.50 | 0.46 |
| Liver cirrhosis | 2.68 | 1.10-6.51 | 0.03 | 1.59 | 0.29-8.65 | 0.59 |
| Chronic kidney disease | 3.10 | 1.17-8.25 | 0.02 | 1.01 | 0.19-5.35 | 0.99 |
| **HIV-related factors** |  |  |  |  |  |  |
| Homosexual male | 0.62 | 0.40-0.95 | 0.03 | 1.57 | 0.68-3.62 | 0.30 |
| Hepatitis B or C coinfection | 0.81 | 0.55-1.21 | 0.31 | - | - | - |
| Duration of HIV infection, per 1-year increase | 1.04 | 1.01-1.08 | 0.02 | 1.01 | 0.85-1.19 | 0.95 |
| Duration of antiretroviral therapy, per 1-year increase | 1.06 | 1.01-1.07 | 0.01 | 0.91 | 0.84-0.99 | 0.03 |
| **History of antiretroviral therapy** | | | | | | |
| Zidovudine | 1.59 | 1.06-2.37 | 0.02 | 0.91 | 0.41-2.03 | 0.82 |
| Abacavir | 1.63 | 1.10-2.42 | 0.02 | 1.30 | 0.67-2.53 | 0.45 |
| Tenofovir | 0.63 | 0.42-0.94 | 0.02 | 1.19 | 0.63-2.24 | 0.59 |
| NNRTI, > 2 years | 0.84 | 0.54-1.29 | 0.42 | - | - | - |
| Unboosted atazanavir, > 2 years | 1.54 | 1.00-2.38 | 0.05 | 1.53 | 0.76-3.06 | 0.23 |
| Atazanavir/ritonavir, > 2 years | 3.61 | 1.21-10.75 | 0.02 | 4.70 | 1.34-16.54 | 0.02 |
| Lopinavir/ritonavir, > 2 years | 1.20 | 0.70-2.04 | 0.51 | - | - | - |
| Darunavir/ritonavir, > 2 years | 1.76 | 0.37-8.39 | 0.48 | - | - | - |
| Indinavir/ritonavir, > 2 years | 3.02 | 1.35-6.72 | 0.01 | 3.96 | 0.87-18.07 | 0.08 |
| **Laboratory investigations** |  |  |  |  |  |  |
| Baseline PVL, per 1 log_10_ copies/mL increase | 1.38 | 0.99-1.89 | 0.50 | - | - | - |
| Baseline CD4 count, per 100-cell/μL decrease | 1.16 | 1.03-1.32 | 0.02 | 1.14 | 0.98-1.33 | 0.10 |
| Follow-up PVL, per 1 log_10_ copies/mL increase | 1.02 | 0.90-1.15 | 0.76 | - | - | - |
| Follow-up CD4 count, per 100-cell/μL decrease | 1.08 | 1.00-1.16 | 0.04 | 1.05 | 0.92-1.20 | 0.47 |
| Estimated GFR,  per 1-mL/min/1.73m^2^ decrease | 1.01 | 1.00-1.02 | 0.003 | 1.00 | 0.99-1.02 | 0.65 |
| Serum total bilirubin, per 1-mg/dL increase | 1.11 | 1.00-1.22 | 0.05 | 1.29 | 1.05-1.57 | 0.01 |
| Serum ALT, per 1-U/L increase | 1.00 | 1.00-1.01 | 0.25 | - | - | - |
| Serum total cholesterol, per 1-mg/dL increase | 1.00 | 1.00-1.01 | 0.77 | - | - | - |
| Serum triglyceride, per 1-mg/dL increase | 1.00 | 1.00-1.00 | 0.37 | - | - | - |

**Abbreviations:** ALT, alanine aminotransferase; CI, confidence interval; GFR, glomerular filtration rate; NNRTI, non-nucleoside reverse-transcriptase inhibitor; NRTI, nucleoside reverse-transcriptase inhibitor; OR, odds ratio; PVL, plasma HIV RNA load.

^a^Variables considered for entry into multivariate logistic regression model included variables with *P* values <0.10 in univariate analysis.

(B) Nephrolithiasis

| **Variable** | **Univariate analysis** | | | **Multivariate analysis^a^** | | |
| --- | --- | --- | --- | --- | --- | --- |
|  | **OR** | **95% CI** | ***P*** | **OR** | **95% CI** | ***P*** |
| **Demographics** |  |  |  |  |  |  |
| Age, per 1-year increase | 1.05 | 1.03-1.07 | 0.001 | 1.03 | 1.00-1.06 | 0.05 |
| Male sex | 2.50 | 0.93-6.67 | 0.07 | 0.95 | 0.18-5.26 | 0.95 |
| Body-mass index , per 1-kg/m^2^ increase | 0.95 | 0.88-1.02 | 0.17 | - | - | - |
| **Underlying diseases** |  |  |  |  |  |  |
| Hyperlipidemia | 1.84 | 1.09-3.09 | 0.02 | 1.14 | 0.52-2.48 | 0.75 |
| Chronic hepatitis | 1.84 | 1.01-3.36 | 0.05 | 1.32 | 0.47-3.69 | 0.60 |
| Hypertension | 2.03 | 1.09-3.77 | 0.03 | 1.48 | 0.58-3.83 | 0.42 |
| Diabetes mellitus | 1.96 | 0.89-4.31 | 0.10 | 1.09 | 0.26-4.63 | 0.91 |
| Liver cirrhosis | 2.77 | 1.01-7.57 | 0.05 | 1.00 | 0.16-6.20 | 0.99 |
| Chronic kidney disease | 3.91 | 1.38-11.06 | 0.01 | 1.65 | 0.38-7.25 | 0.51 |
| **HIV-related factors** |  |  |  |  |  |  |
| Homosexual male | 0.53 | 0.32-0.88 | 0.01 | 0.67 | 0.31-1.45 | 0.31 |
| Hepatitis B or C coinfection | 0.83 | 0.52-1.33 | 0.44 | - | - | - |
| Duration of HIV infection, per 1-year increase | 1.03 | 0.99-1.07 | 0.10 | 0.97 | 0.82-1.15 | 0.73 |
| Duration of antiretroviral therapy, per 1-year increase | 1.04 | 0.99-1.10 | 0.08 | 0.95 | 0.87-1.03 | 0.21 |
| **History of antiretroviral therapy** | | | | | | |
| Zidovudine | 1.22 | 0.74-1.99 | 0.44 | - | - | - |
| Abacavir | 1.44 | 0.89-2.31 | 0.13 | - | - | - |
| Tenofovir | 0.82 | 0.51-1.32 | 0.41 | - | - | - |
| NNRTI, > 2 years | 1.52 | 0.93-2.46 | 0.09 | 1.21 | 0.55-2.68 | 0.64 |
| Unboosted atazanavir, > 2 years | 1.00 | 0.57-1.76 | 0.99 | - | - | - |
| Atazanavir/ritonavir, > 2 years | - | - | 0.99 | - | - | - |
| Lopinavir/ritonavir, > 2 years | 1.00 | 0.52-1.96 | 0.99 | - | - | - |
| Darunavir/ritonavir, > 2 years | - | - | 0.99 | - | - | - |
| Indinavir/ritonavir, > 2 years | 1.69 | 0.57-4.95 | 0.34 | - | - | - |
| **Laboratory investigations** |  |  |  |  |  |  |
| Baseline PVL, per 1-log_10_ copies/mL increase | 1.47 | 0.99-2.16 | 0.13 | - | - | - |
| Baseline CD4 count, per 100-cell/μL decrease | 1.16 | 1.01-1.35 | 0.04 | 1.11 | 0.93-1.33 | 0.23 |
| Follow-up PVL, per 1-log_10_ copies/mL increase | 0.89 | 0.74-1.06 | 0.19 | - | - | - |
| Follow-up CD4 count, per 100-cell/μL decrease | 1.06 | 0.97-1.15 | 0.19 | - | - | - |
| Estimated GFR,  per 1-mL/min/1.73m^2^ decrease | 1.02 | 1.01-1.03 | 0.01 | 1.02 | 1.01-1.03 | 0.01 |
| Serum total bilirubin, per 1-mg/dL increase | 0.94 | 0.76-1.17 | 0.60 | - | - | - |
| Serum ALT, per 1-U/L increase | 1.00 | 1.00-1.01 | 0.12 | - | - | - |
| Serum total cholesterol, per 1-mg/dL increase | 1.01 | 1.00-1.01 | 0.01 | 1.01 | 1.01-1.02 | 0.001 |
| Serum triglyceride, per 1-mg/dL increase | 1.00 | 1.00-1.00 | 0.20 | - | - | - |
| Serum uric acid, per 1-mg/dL increase | 1.01 | 0.73-1.40 | 0.95 | - | - | - |
| Urine pH, per 1-unit increase | 1.41 | 0.89-2.23 | 0.14 | - | - | - |
| Urinary crystal | 2.16 | 0.79-5.87 | 0.13 | - | - | - |

**Abbreviations:** ALT, alanine aminotransferase; CI, confidence interval; GFR, glomerular filtration rate; NNRTI, non-nucleoside reverse-transcriptase inhibitor; NRTI, nucleoside reverse-transcriptase inhibitor; OR, odds ratio; PVL, plasma HIV RNA load.

^a^Variables considered for entry into multivariate logistic regression model included variables with *P* values <0.10 in univariate analysis.
