# Supplementary material for: Cholelithiasis and Nephrolithiasis in HIV-Positive Patients in the Era of Combination Antiretroviral Therapy
Source: PLoS One. 2015 Sep 11;10(9):e0137660. doi: 10.1371/journal.pone.0137660 (PMC4567270; doi:10.1371/journal.pone.0137660)
Supplement: S2 Table — (DOCX) [file pone.0137660.s002.docx]

**Supporting information**

**S2 Table.** Univariate logistic analysis to estimate the factors associated with incident cholelithiasis and nephrolithiasis in 608 antiretroviral-experienced patients.

(A) Cholelithiasis

| **Variable** | **Cholelithiasis** | | |
| --- | --- | --- | --- |
|  | **OR** | **95% CI** | ***P*** |
| **Demographics** |  |  |  |
| Age, per 1-year increase | 1.04 | 1.01-1.07 | 0.01 |
| Male sex | 0.34 | 0.07-1.56 | 0.16 |
| Body-mass index, per 1-kg/m^2^ increase | 1.10 | 0.98-1.23 | 0.11 |
| **Underlying diseases** |  |  |  |
| Hyperlipidemia | 1.24 | 0.52-2.96 | 0.63 |
| Chronic hepatitis | 2.60 | 1.07-6.30 | 0.03 |
| Hypertension | 1.58 | 0.53-4.68 | 0.41 |
| Diabetes mellitus | 2.31 | 0.66-8.04 | 0.19 |
| Liver cirrhosis | 1.33 | 0.17-10.37 | 0.78 |
| Chronic kidney disease | 1.75 | 0.22-13.87 | 0.60 |
| **HIV-related factors** |  |  |  |
| Homosexual male | 0.94 | 0.37-2.35 | 0.89 |
| Hepatitis B or C coinfection | 1.88 | 0.86-4.11 | 0.11 |
| Duration of HIV infection, per 1-year increase | 1.04 | 0.98-1.10 | 0.18 |
| Duration of antiretroviral therapy, per 1-year increase | 1.06 | 0.98-1.15 | 0.13 |
| **History of antiretroviral therapy** |  |  |  |
| Zidovudine | 1.09 | 0.51-2.35 | 0.83 |
| Abacavir | 1.97 | 0.88-4.39 | 0.10 |
| Tenofovir | 0.68 | 0.32-1.43 | 0.31 |
| NNRTI, > 2 years | 1.03 | 0.48-2.22 | 0.93 |
| Unboosted atazanavir, > 2 years | 2.05 | 0.96-4.37 | 0.07 |
| Atazanavir/ritonavir, > 2 years | 7.40 | 1.92-28.49 | 0.004 |
| Lopinavir/ritonavir, > 2 years | 1.28 | 0.51-3.22 | 0.60 |
| Darunavir/ritonavir, > 2 years | - | - | 0.99 |
| Indinavir/ritonavir, > 2 years | 1.02 | 0.13-7.85 | 0.98 |
| **Laboratory investigations** |  |  |  |
| Baseline PVL, per 1-log_10_ copies/mL increase | 2.07 | 1.08-3.98 | 0.03 |
| Baseline CD4 count, per 100-cell/μL decrease | 1.15 | 0.91-1.47 | 0.24 |
| Follow-up PVL, per 1-log_10_ copies/mL increase | 1.43 | 1.08-1.88 | 0.01 |
| Follow-up CD4 count, per 100-cell/μL decrease | 1.08 | 0.94-1.25 | 0.27 |
| Estimated GFR, per 1-mL/min/1.73m^2^ decrease | 1.01 | 1.00-1.03 | 0.12 |
| Serum total bilirubin, per 1-mg/dL increase | 1.35 | 1.06-1.71 | 0.02 |
| Serum ALT, per 1-U/L increase | 1.01 | 1.00-1.01 | 0.09 |
| Serum total cholesterol, per 1-mg/dL increase | 1.00 | 0.99-1.01 | 0.77 |
| Serum triglyceride, per 1-mg/dL increase | 1.00 | 1.00-1.00 | 0.76 |

**Abbreviations:** ALT, alanine aminotransferase; CI, confidence interval; GFR, glomerular filtration rate; NNRTI, non-nucleoside reverse-transcriptase inhibitor; NRTI, nucleoside reverse-transcriptase inhibitor; OR, odds ratio; PVL, plasma HIV RNA load*.*

(B) Nephrolithiasis

| **Variable** | **Nephrolithiasis** | | |
| --- | --- | --- | --- |
|  | **OR** | **95% CI** | ***P*** |
| **Demographics** |  |  |  |
| Age, per 1-year increase | 1.05 | 1.02-1.08 | 0.004 |
| Male sex | 0.29 | 0.06-1.33 | 0.11 |
| Body-mass index, per 1-kg/m^2^ increase | 0.93 | 0.82-1.06 | 0.28 |
| **Underlying diseases** |  |  |  |
| Hyperlipidemia | 2.25 | 0.97-5.21 | 0.06 |
| Chronic hepatitis | 1.99 | 0.73-5.47 | 0.18 |
| Hypertension | 3.25 | 1.25-8.46 | 0.02 |
| Diabetes mellitus | 0.79 | 0.10-5.98 | 0.82 |
| Liver cirrhosis | 1.56 | 0.20-12.24 | 0.67 |
| Chronic kidney disease | 4.66 | 0.99-22.03 | 0.05 |
| **HIV-related factors** |  |  |  |
| Homosexual male | 0.51 | 0.21-1.20 | 0.12 |
| Hepatitis B or C coinfection | 2.56 | 1.06-6.22 | 0.04 |
| Duration of HIV infection, per 1-year increase | 1.06 | 1.01-1.12 | 0.03 |
| Duration of antiretroviral therapy, per 1-year increase | 1.10 | 1.02-1.20 | 0.02 |
| **History of antiretroviral therapy** | | | |
| Zidovudine | 1.41 | 0.63-3.16 | 0.40 |
| Abacavir | 2.83 | 1.12-7.17 | 0.03 |
| Tenofovir | 0.57 | 0.25-1.27 | 0.17 |
| NNRTI, > 2 years | 2.22 | 0.99-4.96 | 0.05 |
| Unboosted atazanavir, > 2 years | 0.88 | 0.35-2.24 | 0.79 |
| Atazanavir/ritonavir, > 2 years | - | - | 0.99 |
| Lopinavir/ritonavir, > 2 years | 1.23 | 0.45-3.33 | 0.69 |
| Darunavir/ritonavir, > 2 years | - | - | 0.99 |
| Indinavir/ritonavir, > 2 years | 1.20 | 0.16-9.27 | 0.86 |
| **Laboratory investigations** |  |  |  |
| Baseline PVL, per 1-log_10_ copies/mL increase | 1.59 | 0.75-3.37 | 0.23 |
| Baseline CD4 count, per 100-cell/μL decrease | 1.33 | 0.93-1.89 | 0.11 |
| Follow-up PVL, per 1-log_10_ copies/mL increase | 0.78 | 0.41-1.46 | 0.43 |
| Follow-up CD4 count, per 100-cell/μL decrease | 1.09 | 0.93-1.27 | 0.30 |
| Estimated GFR, per 1-mL/min/1.73m^2^ decrease | 1.01 | 0.99-1.03 | 0.17 |
| Serum total bilirubin, per 1-mg/dL increase | 1.10 | 0.85-1.41 | 0.48 |
| Serum ALT, per 1-U/L increase | 1.00 | 0.99-1.01 | 0.65 |
| Serum total cholesterol, per 1-mg/dL increase | 1.01 | 1.00-1.02 | 0.01 |
| Serum triglyceride, per 1-mg/dL increase | 1.00 | 1.00-1.00 | 0.58 |
| Serum uric acid, per 1-mg/dL increase | 0.57 | 0.12-2.67 | 0.47 |
| Urine pH, per 1-unit increase | 2.07 | 0.89-4.84 | 0.09 |
| Urinary crystal | - | - | 0.99 |

**Abbreviations:** ALT, alanine aminotransferase; CI, confidence interval; GFR, glomerular filtration rate; NNRTI, non-nucleoside reverse-transcriptase inhibitor; NRTI, nucleoside reverse-transcriptase inhibitor; OR, odds ratio; PVL, plasma HIV RNA load*.*
