# Supplementary material for: Cholelithiasis and Nephrolithiasis in HIV-Positive Patients in the Era of Combination Antiretroviral Therapy
Source: PLoS One. 2015 Sep 11;10(9):e0137660. doi: 10.1371/journal.pone.0137660 (PMC4567270; doi:10.1371/journal.pone.0137660)
Supplement: S1 Text — (DOCX) [file pone.0137660.s003.docx]

**Supporting information**

**S1 Text.** The detailed methods for determination of plasma atazanavir concentrations and genetic polymorphisms.

***Determination of plasma atazanavir concentrations***

The amount of 300 μL of plasma was added to 300 mL of acetonitrile for deproteinization, and then the organic layer was dried under nitrogen. The extract was dissolved with 150 mL of methanol and 200 mL of 10 mM phosphate buffer (pH 4.0) mixed with acetonitrile in the ratio of 57:43 (volume/volume). The HPLC system was composed of a L-2130 HTA solvent delivery pump, a L-2200 autosampler, a UV1000 wavelength detector programmable UV detector wavelength 245 nm and the computing integrator for HPLC D-2000 Elite on Windows (version 1.2, Hitachi High Technologies Corporation, Tokyo, Japan). Chromatography was performed on the C18 column (Mightsil RP-18 GP, 25064.6 nm with 5 mm beads, particle size 3.5 mm; Kanto Corporation, Portland, OR, USA) protected by a guard column (1033 mm I.D.; Phenomenex, Torrance, CA, USA), and with a flow rate of 1 mL/min. The mobile phase was consisted of aforementioned phosphate buffer mixed with acetonitrile. The retention time of atazanavir was 3.7 min. The calibration curve was linear within the range 0.5 to 10.0 mg/L, and data more than 10.0 mg/L was designated as 10.0 mg/L. The lower limit of quantification was 0.15 mg/L. Recovery after extraction from plasma was 101%. Accuracy ranged from 97.7 to 101.6%. Intra-assay and inter-assay coefficients of variation at 5 mg/mL ranged from 0.2 to 0.97% and 1.15 to 1.93%, respectively.

***Genetic polymorphisms***

DNA amplification was performed under similar conditions for each reaction with the use of PCR buffer (Invitrogen, Carlsbad, CA, USA) with 5 pmol of specified primers (Mission Biotech, Taiwan), 0.2 mmol/L of deoxyribonucleoside triphosphate (Promega, WI, USA), 2 mmol/L of magnesium chloride, and 0.5 units of Platinum Taq (Invitrogen, Carlsbad, CA, USA) in a total volume of 25 μL. PCR amplification comprised an initial denaturation for 2 minutes at 94°C, followed by 35 cycles of denaturation at 94°C for 30 seconds, annealing at 60°C for 30 seconds, extension at 72°C for 30 seconds, and terminal elongation at 72°C for 7 minutes. DNA fragments generated after restriction enzyme digestion were separated on a 3.5% agarose gel and visualized after ethidium bromide staining of the agarose gel with the use of an ultraviolet transilluminator.
